# Epidemiology of adult ankle fractures in Sweden between 1987 and 2004: A population-based study of 91,410 Swedish inpatients

**DOI:** 10.3109/17453674.2012.672091

**Published:** 2012-06-04

**Authors:** Charlotte K Thur, Gustaf Edgren, Karl-Åke Jansson, Per Wretenberg

**Affiliations:** ^1^Department of Molecular Medicine and Surgery, Section of Orthopaedics and Sports Medicine, Karolinska Institutet and Karolinska University Hospital; ^2^Department of Medical Epidemiology and Biostatistics, Karolinska Institutet, Stockholm, Sweden, and Department of Epidemiology, Harvard School of Public Health, Boston, MA, USA

## Abstract

**Background and purpose:**

Previous national epidemiological data on the characteristics and trends of patients with ankle fractures have been limited. We therefore analyzed data on Swedish inpatients with ankle fractures in this nationwide population study, based on data from 1987 through 2004.

**Patients and methods:**

Data on all inpatients aged 15 years and older with ankle fracture were extracted from the Swedish National Patient Register for the period 1987–2004.

**Results:**

We identified 91,410 hospital admissions with ankle fracture, corresponding to an annual incidence rate of 71 per 10^5^ person-years. During the study period, the number of hospital admissions increased by 0.2% annually, mainly from increase in fracture incidence in the elderly women. Mean age at admission was 45 (SD 19) years for men and 58 (18) for women. The major mechanism of injury was falling at the same level (64%).

**Interpretation:**

This nationwide study of inpatients with ankle fractures showed an increase in fracture incidence, particularly in elderly women.

Ankle fractures are among the most common fractures treated in orthopaedic surgery today. They are also a significant source of morbidity in both the young and the elderly (Donaldson et al. 1990, Jones et al. 1994, van Staa et al. 2001). Previous epidemiological studies have shown trends of increasing incidence over time, mainly in elderly women (Bengnér et al. 1986, Daly et al. 1987, Baron et al. 1996, Kannus et al. 1996, Court-Brown et al. 1998, van Staa et al. 2001, Kannus et al. 2002). However, most of these studies have included limited numbers of patients, and mostly originated from single hospitals or limited areas.

Basic epidemiological data such as incidence, fracture type, age and sex distribution, mechanisms of injury, and surgical procedures can provide estimates when discussing disease burden and in the planning and provision of healthcare.

The purpose of this study was to perform an epidemiological analysis of all adult ankle fractures requiring hospital admission in Sweden from 1997 through 2004, including incidence, causes of fracture, surgical procedures, patient characteristics, and trends over time.

## Patients and methods

### Source of data

The study was based on the Swedish National Patient Register which has recorded data on individual hospital discharges and surgical procedures since 1964, with nationwide coverage since 1987. The register has been found to capture more than 98% of all hospital admissions in Sweden (Socialstyrelsen 1, Socialstyrelsen 2, Ludvigsson et al. 2011). The information recorded in the register includes a unique 10-digit personal identification number, which identifies individuals uniquely and allows unbiased linkage between registers, and also dates of hospital admission and discharge, diagnosis at discharge, and surgical procedures.

The register also provides data on age, sex, and medical diagnoses. The validity of the data has recently been shown to be very high (Ludvigsson et al. 2011). Discharge diagnoses are coded according to a Swedish adaptation of the International Classification of Diseases (ICD), revisions 7–10, and surgical procedures according to the Swedish version of Classification of Surgical Procedures. During the study period, the ICD coded register was revised; ICD-9 was used between 1987 and 1996, and ICD-10 was used from 1997 to the present. One county, Skåne, used the ICD-9 version throughout 1997 and changed to ICD-10 in 1998.

Using the ICD, we identified the relevant codes for malleolar fractures: lateral malleolar fracture closed and open (824C/824D (ICD9) S82.60/61 (ICD10)), medial malleolar fracture closed and open (824A/824B (ICD9) S82.50/51 (ICD10)) and bi- and tri-malleolar fracture closed and open (824E/824F/824G/824H (ICD9) S82.80/81 (ICD10)).

External causes of fracture were classified according to ICD external codes (E-codes) and grouped into 6 categories: fall at the same level, fall from a height, fall unspecified, transport accident, miscellaneous, and not reported (i.e. no E-code had been reported to the register).

Surgical interventions were identified by using procedure codes related to ankle fracture surgery and grouped into 5 categories: open reduction and internal osteosynthesis, closed reduction and external support, external fixation (Hoffman), miscellaneous, and not reported.

To account for possible surgical delay, all surgical procedures recorded within 30 days of first admission for an ankle fracture were considered. The procedure code register was revised during the study period (1997) (Socialstyrelsen 3, Socialstyrelsen 4).

To allow calculation of incidence, we also obtained data on the age-, sex- and calendar period-specific size of the entire Swedish population, as provided by the total population register maintained by Statistics Sweden (Ludvigsson et al. 2011).

The study was approved by the local Ethics Committee (DN 2006/156-31).

### Study population and statistical analysis

We identified all patients who were recorded to have been discharged from hospital with a relevant discharge diagnosis between January 1, 1987 and December 31, 2004. We excluded patients younger than 15 years, as this is the cutoff age commonly used in Sweden for adult fractures. Also, as we only wanted to study first occurrence of fractures, all readmissions of the same patient after primary hospitalization were ignored. All hospitalizations were then classified according to age, sex, type of fracture, type of surgical procedure, and length of hospital stay.

Differences in means were assessed using t-tests, assuming equal or unequal variances as appropriate, based on an F-test for the equality of variance. In all cases the variances were found to be unequal, and Satterthwaite's method for t-test was used. Age-, sex-, calendar period-, and fracture type-specific incidence rates of ankle fractures for the Swedish population, between 1987 and 2004, were calculated by dividing the number of admissions for the specified fractures by the corresponding end-of-year age-, sex-, and calendar period-specific size of the background population. Mid-year population size was estimated by taking the geometric mean of year-end population sizes of consecutive years. Confidence intervals were constructed for incidence rates based on the assumption that the number of fractures followed a Poisson distribution (Rosner 2006). Changes in length of hospital stay were assessed using univariate linear regression. Closed and open fractures were analyzed separately. To assess changes in incidence, we fitted multiple Poisson regression models which allowed multivariate adjustment for age, sex, and calendar period. All variables were treated as categorical: age in 5-year categories between 15 and 100 and calendar period in yearly categories between 1987 and 2004. Tests of incidence trends were conducted by including the relevant variables as linear terms in the Poisson model. All p-values < 0.05 were considered to be statistically significant.

All statistical analyses were performed with the statistics package SAS System 9.2 (SAS Institute Inc., Cary, NC).

## Results

### Admissions and patients

Over the 18-year study period (1987–2004), 91,410 patients were recorded to have sustained an ankle fracture that required hospital admission. Of these, 51,700 (57%) were women. The overall mean age was 52 (SD 20). Men were on average younger than women, 45 (19) vs. 58 (19) years (p < 0.001). The commonest fracture types in women were bi-/tri-malleolar (57%) and in men they were lateral malleolar (49%). 97% of the fractures were closed (Table 1).

**Table 1.** Descriptive data on 91,410 ankle fractures in Sweden during the period 1987–2004

	Women		Men	
Number, n (%)	51,700	57%	39,710	43%
Closed fracture (%)	50,066	97%	38,391	97%
Open fracture (%)	1,634	3%	1,319	3%
Age at hospital admission, n (%)				
15–30	4,384	8%	9,854	25%
30–39	4,557	9%	6,371	16%
40–49	7,472	14%	6,891	17%
50–59	10,115	20%	6,638	17%
60–69	9,646	19%	4,608	12%
70–79	8,710	17%	3,455	9%
≥ 80	6,816	13%	1,893	5%
Mean age (SD)	58	(19)	45	(19)
Closed fracture	58	(19)	45	(19)
Open fracture	59	(18)	47	(19)
Type of fracture, n (%)				
Medial malleolar	3,299	6%	4,821	12%
Lateral malleolar	18,917	37%	19,572	49%
Bi-/tri-malleolar	29,484	57%	15,317	39%

### Incidence rates

**Figure 1.**
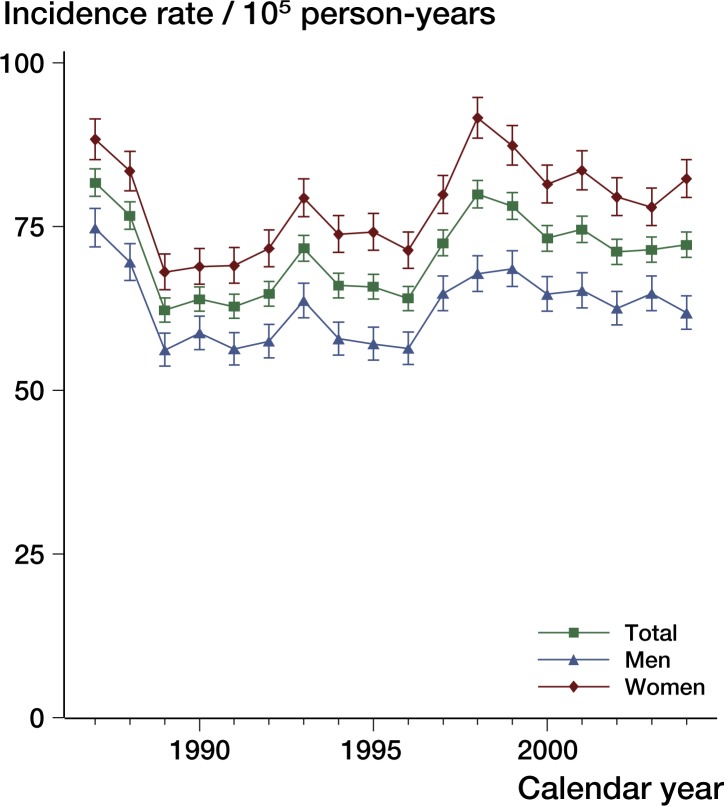
Incidence rates (per 10^5^ person-years with 95% CI) of ankle fractures in Sweden during the period 1987–2004, stratified by sex and year.

The total crude incidence rate was 71 (95% confidence interval (CI): 70–71) per 10^5^ person-years. Men had an incidence rate of 63 (CI: 62–63) per 10^5^ person-years and women had an incidence rate of 79 (CI: 78–79) per 10^5^ person-years. Poisson regression analysis showed that the overall incidence rate increased by 0.2% per year (p < 0.001). The incidence rate for men increased by 0.1%, while the incidence for women increased by 0.3% (p < 0.001) (Figure 1). In elderly women over 60 years of age, the incidence increased by 0.9% per year over the study period (p < 0.001).

**Figure 2.**
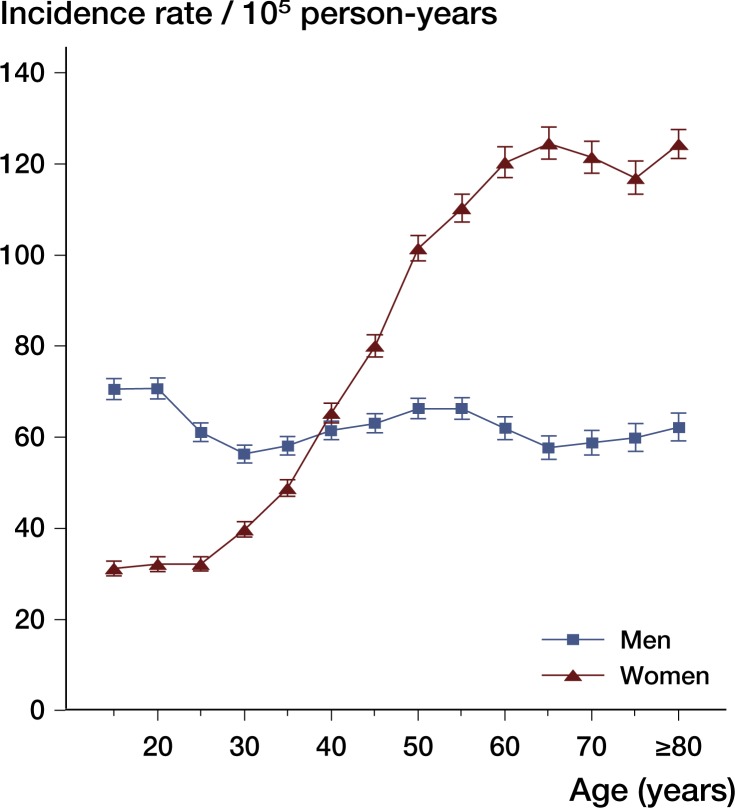
Incidence rates (per 10^5^ person-years with 95% CI) of ankle fractures in Sweden during the period 1987–2004, stratified by age and sex.

**Figure 3.**
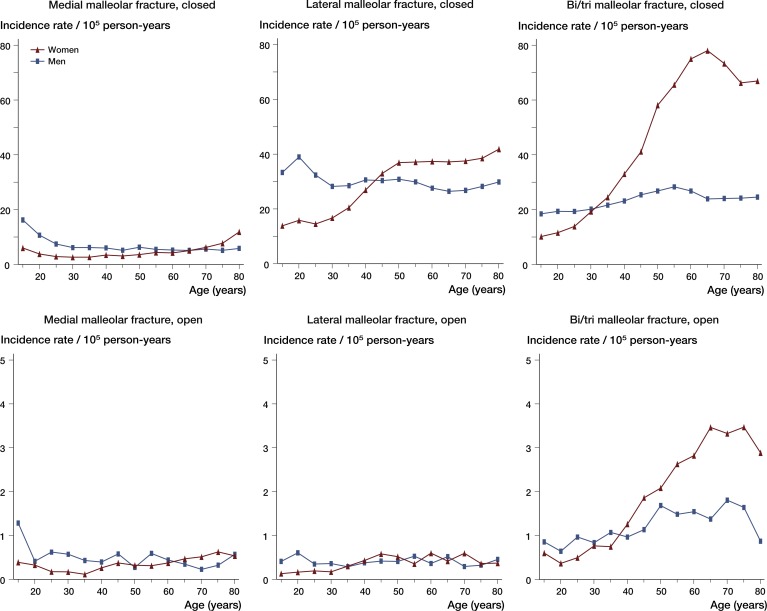
Incidence rates (per 10^5^ person-years) of ankle fractures in Sweden during the period 1987–2004, stratified by age, sex, and type of fracture. Note the different scale for open fractures.

Men had their peak incidence during the first decades of life (age 15–29), at 67 (CI: 66–68) per 10^5^ person-years, while women had an increase in fracture incidence from their fifth decade of life, at 107 (CI: 104–109) per 10^5^ person-years and increasing with age (Figure 2). When broken down by fracture type, it was evident that the total was dominated by closed bi- or tri-malleolar fractures, at 33 (CI: 33–34) per 10^5^ person-years, followed by closed lateral malleolar fractures, at 29 (CI: 29–30) per 10^5^ person-years. The incidence of closed bi- and tri-malleolar fractures was most pronounced in elderly women (Figure 3).

### Mechanism of injury

The most common mechanism of injury for all fractures was a fall at the same level (64%), followed by a fall from height (10%) (Table 2). In women over 60 years, falling at the same level accounted for 72% of cases.

**Table 2.** Mechanisms of injury

	Women	(%)	Men	(%)	Closed fractures	(%)	Open fractures	(%)	Total	(%)
Fall at same level	35,829	69	22,372	56	56,834	64	1,367	46	58,201	64
Fall from height	5,089	10	3,676	9	8,383	10	382	13	8,765	10
Fall unspecified	2,778	6	1,985	5	4,665	5	98	3	4,763	5
Transport accident	3,564	7	4,604	12	7,591	9	577	20	8,168	9
Miscellaneous	2,692	5	5,828	15	8,111	9	409	14	8,520	9
Not reported	1,748	3	1,245	3	2,873	3	120	4	2,993	3

Open fractures were caused by transport accidents in 20% of admissions, as compared to 9% in the closed fracture group. The incidence of fractures from transport accidents decreased by an average of 1.3% per year (p < 0.001) during the study period.

### Surgical procedures

83% of all admissions resulted in some kind of surgical procedure, with a slightly higher number for men (84%) than for women (81%). The surgical procedure was dominated by some kind of open reduction and internal fixation (76%). Analyses stratified by sex revealed that 78% of the male admissions resulted in open reduction and internal osteosynthesis, as compared to 75% in women. Comparing open and closed fractures, 76% resulted in open reduction and internal osteosynthesis in both groups. 2% of the open fracture group did have some kind of external fixation during hospital admission, as compared to < 1% (0.2%) for the closed fractures (Table 3).

**Table 3.** Surgical procedures (within 30 days of first admission)

	Women	(%)	Men	(%)	Closed fractures	(%)	Open fractures	(%)	Total	(%)
Open reduction/int. osteosynthesis	38,820	75	30,990	78	67,560	76	2,250	76	69,810	76
Closed reduction/ext. support	2,852	5	2,110	5	4,846	6	116	4	4,962	5
External fixation (Hoffman)	116	< 1	111	< 1	174	< 1	53	2	227	< 1
Miscellaneous	284	< 1	217	< 1	472	< 1	29	1	501	< 1
Not reported	9,628	19	6,282	16	15,405	17	505	17	15,910	17

### Length of hospital stay

Patients were hospitalized for mean 6.6 (SD 6.6) days. The mean length of hospital stay for closed fractures was 6.5 (6.6) days and for open fractures it was 9.2 (8.4) days (p < 0.001). Men were hospitalized for a shorter time than women on average, 5.7 (5.9) days and 7.2 (7.1) days, respectively (p < 0.001). Linear regression analysis revealed that the average length of stay decreased by 0.16 days per year in the closed fracture group (p < 0.001). In the open fracture group, the average length of stay decreased by 0.14 days per year (p < 0.001) during the study period.

For women over 60 years, the mean length of stay was 10.1 (SD 8.6) days. The type of fracture that resulted in the longest length of stay was the open bi-/tri-malleolar fracture, at 10.0 (8.5) days.

## Discussion

This study involves one of the largest numbers of ankle fractures published so far, and more importantly, represents more than just a random sample. The study covered close to 100% of all adult patients admitted to hospital with an ankle fracture in Sweden during the period 1987–2004. As in previous studies (Bengnér et al. 1986, Court-Brown et al. 1998), we found an increase in ankle fractures during the study period. At 0.2% per year, the increase was, however, lower than previously reported. There may be different reasons for our findings. They may be due to a true reduced increase in fracture incidence over the period. They might also be due to a shift from inpatient treatment to outpatient treatment. From 1992 to 2002, the number of hospital beds in Sweden decreased by 47% (Molin and Johansson 2004), and hospital care has shifted from inpatient care to outpatient care. Our study did not include outpatient care, since that part of the register started in 2001 and the coverage is considerably lower (about 80%) (Ludvigsson et al. 2011).

We also found a lower total incidence of ankle fractures in this study than previously reported. Bengnér et al. (1986) found an increase from about 65 per 10^5^ person-years to about 107 per 10^5^ person-years over a 30-year period (1950–1980). A decade later, 1988–1990, Court-Brown et al. (1998) found an incidence of 122 ankle fractures per 10^5^ person-years. An even higher incidence was found by Daly et al. (1987), at 187 per 10^5^ person-years. Unlike the present study, their study included both avulsion fractures and fractures in children. These studies all had larger incidence rates than our study since both inpatients and outpatients were included.

The increase in incidence was most pronounced in women over 60 years. An epidemiological study by Kannus et al. (2002) showed an increase in incidence in women over 60 years of 164% in the years 1970–2000. A later study by the same author did, however, show a stabilizing and slightly decreasing fracture incidence rate for this group from 2000 to 2006 (Kannus et al. 2008). In the present study, we had a more constant increase (an average of 1% per year) for this group.

Consistent with several previous studies (Bengnér et al. 1986, Daly et al. 1987, Court-Brown et al. 1998, Jensen et al. 1998, van Staa et al. 2001), we found an increasing incidence of ankle fractures in women. This increase was mainly seen after the fourth decade of life, which is comparable with the findings of Johansen et al. (1997), who reported a rise in incidence for any type of fracture at this age. A few of the same studies have, however, reported a decrease in incidence of ankle fracture in women after the age of 60 (Bengnér et al. 1986, Daly et al. 1987, Jensen et al. 1998). This contrasts with our results, where these rates remained twice as high as for men. The reason for this is not known, but it could be explained by an ageing but still active population (Court-Brown and Caesar 2006).

Whether or not an ankle fracture in the elderly is an osteoporotic fracture can be discussed (Court-Brown and Caesar 2006). Previous studies have not shown any decrease in bone mineral density (BMD) in ankle fracture patients (Seeley et al. 1991, Greenfield and Eastell 2001, Hasselman et al. 2003). Despite this, there was a clear dominance of the more unstable bi- and tri-malleolar fractures in elderly women, similar to that in previous studies (Bengnér et al. 1986, Court-Brown et al. 1998). This is of importance, since the elderly population have poorer health preoperatively (based on the number of comorbidities) and have higher perioperative complication rates compared to younger patients (Anderson et al. 2008). It is also known that bi- and tri-malleolar fractures have a higher mortality rate than uni-malleolar fractures (Koval et al. 2007). These fractures are a challenge, both surgically and concerning perioperative care.

The average length of stay was in accordance with that in a previous study by Jensen et al. (1998). Daly et al. (1987) reported an average length of stay of 9 days, but that only included inpatients treated with open reduction and internal osteosynthesis. There was a trend of reduced length of stay, which is most probably explained by the shift from inpatient care to outpatient care, and is in accordance with the general trend in Sweden during the period (Molin and Johansson 2004).

Open ankle fractures, which are a challenge concerning soft tissue management, had their highest incidence in women aged 60 years and older with bi- and tri-malleolar fractures. In total, the open fracture group accounted for 3% of fractures, which is in accordance with previous studies (Court-Brown et al. 1998). Due to the severity of the injury, length of hospital stay was longer than for closed fractures, but it did also decrease during the study period. As expected, open fractures were more likely to be associated with transport accidents than closed fractures. Transport accidents as the mechanism of injury decreased slightly during the study period, which is in contrast to recent figures (for 1998–2008), where fractures in arms and legs have generally increased somewhat (Transport Analysis).

Most fractures in our study were treated with surgery, as could be expected since we only studied inpatients. Because of this, our rate of surgical procedures was higher than has been reported previously (Daly et al. 1987, Jensen et al. 1998). External fixation was more commonly used with open fractures than with closed fractures. This is not surprising, since open fractures more often need primary damage control.

A limitation of our study is that it included 2 versions of the ICD (ICD9 and ICD10). Using the conversion guides, we could not see any great differences and we believe that a long period of study was an advantage. Also, the design of this study did not differentiate between the lengths of stay before and after surgery. Etiology of fractures is multifaceted and complex, making fracture studies difficult. Despite the extensive coverage of the Swedish National Patient Register, there is still a need for additional variables such as risk factors, earlier comorbidities, and laterality (Ludvigsson et al. 2011).

In conclusion, we have reported age-, sex-, and fracture-specific incidence rates for all ankle fractures admitted between 1987 and the end of 2004 in Sweden. We found a slight increase in the incidence of ankle fractures that were admitted during this period, which was most pronounced for the bi- and tri-malleolar fractures in elderly women.
